# Nursing Attitudes Questionnaire: Testing the Psychometric Characteristics of the Italian Version (NAQ-IV)

**DOI:** 10.3390/healthcare12141366

**Published:** 2024-07-09

**Authors:** Ivan Rubbi, Luana Conte, Gianandrea Pasquinelli, Paola Ferri, Elsa Vitale, Roberto Lupo, Valeria Cremonini

**Affiliations:** 1 School of Nursing, University of Bologna, 48018 Faenza, Italy; ivan.rubbi2@unibo.it (I.R.); valeria.cremonini@auslromagna.it (V.C.); 2 Laboratory of Biomedical Physics and Environment, Department of Mathematics and Physics “E. De Giorgi”, Università del Salento, 73100 Lecce, Italy; 3 Advanced Data Analysis in Medicine (ADAM), Laboratory of Interdisciplinary Research Applied to Medicine (DReAM), Local Health Authority (ASL) Lecce and Università del Salento, 73100 Lecce, Italy; 4 Department of Medical and Surgical Sciences, University of Bologna, 40126 Bologna, Italy; gianandr.pasquinelli@unibo.it; 5 Department of Biomedical, Metabolic and Neural Sciences, University of Modena and Reggio Emilia, 41125 Modena, Italy; paola.ferri@unimore.it; 6 Local Health Authority (ASL) of Bari, 70126 Bari, Italy; vitaleelsa00@gmail.com; 7 ”San Giuseppe da Copertino” Hospital, Local Health Authority (ASL) of Lecce, 73043 Copertino, Italy; robertolupo_2015@libero.it

**Keywords:** Nursing Attitudes Questionnaire, NAQ, NAQ-IV, Italian version, validation

## Abstract

Introduction: The image of a nurse is a source of concern due to its impact on recruitment into the profession, political decisions about the profession, and how the image affects nursing practice. For these reasons, one of the long-term challenges is to assess and maintain a favorable public image that respects the utility and value of the nursing profession. Aim: This study aims to validate an instrument for assessing the image of the nurse as perceived by Italian citizens. Methods: A non-probabilistic sample of 564 people participated in the study between 2022 and 2023. Sociodemographic information of the Italian citizens was collected, and the instrument used to evaluate the perceived public image was the Nursing Attitudes Questionnaire (NAQ). The psychometric properties of the Italian version of the NAQ (NAQ-IV) were calculated using Cronbach’s alpha, item–total correlations, skewness, and kurtosis. Factor analysis was performed using principal axis factoring and the varimax rotation method. Results: Factor analysis revealed a four-factor model explaining more than 60.52% of the variance, with the largest variance explained by the “Role and Professionalism” factor (34.08%). The internal consistency calculation showed a Cronbach’s alpha of 0.89 for the scale and between 0.88 and 0.89 among the factors; all the items verified the item–total correlation and response variability criteria. Conclusions: The NAQ-IV could be a valid tool for assessing the perception of Italian citizens. However, further studies are recommended to evaluate the reliability of the instrument, especially in the evolving professional profile and social health welfare.

## 1. Introduction

Since the time of Florence Nightingale, the public image of nurses has been one of the main challenges for the profession [1]. People’s perception of nursing often depends on their views regarding the roles, values, and professional activities of nurses, and the responsibilities that nurses have towards society [2]. The nursing profession has evolved significantly in recent years [3], with nurses constituting a dominant part of the healthcare system both in terms of quantity and the nature of their roles [4]. However, despite the rapid advancements in education, research, and clinical and organizational competence [5], the public image of nurses has not kept pace [6]. The media image of nurses is a source of concern due to its impact on recruitment into the profession, patient satisfaction and quality of care, nurse motivation and job performance, quality of work life, intention to leave the profession, healthcare policies, and funding for nursing services [5,7]. Although research plays a significant role, the public image of nurses is influenced by other factors, including mass communication, culture, stereotypes, and nurses’ self-esteem [8,9,10,11,12]. Therefore, the scientific community has agreed on the importance of conducting studies that evaluate the public perception of nursing [7], as it has been shown that there is a direct correlation between the positive perception of the profession and the quality of nursing care [4,13,14]. Since 2020, studies aimed at evaluating the image of nurses have significantly increased, recognizing the key role of nurses during the COVID-19 pandemic [8,15,16,17,18,19]. With nurses being on the frontline alongside patients during the pandemic, the public’s perception on social media seems to portray an image of nurses that reflects the professionalism and values of the profession [20]. It is no coincidence that nurses have been represented with artistic images depicting the values of courage and professional dedication in the service of life. The results of some Italian studies in 2023 showed an improvement in the public image compared to previous studies, probably due to the influence of the mass media and the pandemic experience. However, despite these results, the attractiveness of the nursing profession in Italy remains quite low [21]. According to the 2023 Italian Health Report, to develop the territory according to the National Recovery and Resilience Plan (PNRR), between 40,000 and 80,000 nurses are needed, but finding them is currently difficult; the attractiveness of the profession is low, and only 1% of students choose this degree course compared to an average of 3% in other EU countries [22]. In light of these data, the National Federation of Nursing Orders had proposed actions to the Italian government to address the nursing shortage, including improving postgraduate training with clinical specialization master’s degrees, greater recognition of nurses within organizations, and a remuneration system specific to the role [23]. For these reasons, one of the long-term challenges for nursing is to assess and maintain a favorable public image that respects the utility and value of the profession [5].

The Nursing Attitudes Questionnaire (NAQ) is an instrument that assesses people’s perceptions of the nursing profession [2]. The NAQ was adapted by Toth et al. [2] from a previous instrument developed by Hoskins [24]. The validity and reliability of the instrument have been tested in previous studies [25,26].

It is a Likert scale instrument composed of 30 items, with five points, where one means strongly disagree and five means strongly agree. The NAQ scores therefore range from 30 to 150 points, where higher scores indicate a more favorable attitude towards nursing care, while lower scores reflect a less positive perception. Seven items are recoded before statistical analysis [2].

The instrument measures attitudes towards nurses using statements that reflect the roles of the professional, including values, responsibilities, characteristics of nurses/nursing, professionalism, and societal stereotypes. A panel of experts supported the content validity of this instrument, while construct validity was established using the contrastive group approach. Cronbach’s alpha ranged from 0.75 to 0.80 in previous studies [2]. The original language instrument was tested among the population of nursing students, and the calculations were determined on each individual item and not by domain. The content validity for each domain was supported by a group of experts, who added 12 items to a previous instrument called the “Hoskins Questionnaire” [24]. Neither in the study by Toth et al. [2] nor in subsequent studies were the factor analysis calculations highlighted [2,27,28,29].

In Italy, the instrument has been tested with a sample from the entertainment world (VIP) [30], on high school students during orientation meetings for university access, and specifically to the Nursing Degree Course [31], and in the post-COVID-19 pandemic period on the Italian population from North to South Italy [21]. Italian studies used the NAQ in its original form and, like previous studies [2], the areas were determined based on the work of a panel of experts and supported by internal consistency analysis. No factor analysis calculations were performed; only internal consistency was analyzed (α = 0.89) [21,30].

The aim of this paper is to provide literature with an Italian instrument that evaluates the public image of the nursing profession as perceived by ordinary people who are not professionals. For this reason, interviews were conducted with Italian citizens using survey-like methods.

The finality is to validate the Italian version of the NAQ and test its psychometric properties on the Italian population.

## 2. Methods

### 2.1. Translation Procedures

To establish the content validity of the NAQ scale, a forward–back translation procedure was applied. The White and Elander criteria were used as in the pilot study [30]. Firstly, the NAQ scale was translated into Italian and submitted to a panel of experts (5 nurses with expertise in nursing education and research) who compared the original English version of the scale with the Italian version and ensured the semantic and cultural consistency of the items. Secondly, an English lecturer translated the Italian version into English as a blind. Finally, the back-translated and the original instrument were compared by a native speaker.

The reliability of the NAQ has been tested in previous studies [2,27,28,29]. However, these studies used scores from the entire NAQ to compare demographic data or interventions. No factor analysis was performed to test the construct validity. It is unclear which items contribute to which factor or dimension of the concept, “attitude towards nursing care” [29].

### 2.2. Sampling Procedures

Adults from heterogeneous professions were included in the study. In contrast with previous studies [2,27,28,29], the sampling was collected in Italy and the questionnaire was completed by citizens from the north to the south of the country.

### 2.3. Sample Description

A non-probabilistic sample of 564 individuals voluntarily participated in the research. The validation study included people interviewed in the post-pandemic period across the entire Italian territory. The data were extracted from a database of 1345 observations collected from 2017 to January 2023. However, to avoid biases contingent on the changed public perception of nurses engaged in the fight against COVID-19, only questionnaires from the post-pandemic period (August 2022 to January 2023) were considered.

### 2.4. Collection Procedures and Additional Variables

The research project that activated the studies on the image of the nurse was authorized by the Unibo Bioethics Committee on 8 February 2017 prot. 13221. Sampling was collected on a voluntary basis after explaining the purpose of the studies. Anonymity is guaranteed, and there is no information in the database that can be traced back to the identity of the sample. Documents and access to the data were only granted to the author responsible for the research projects. The questionnaires were completed on an online platform in self-administration mode. The respondents were given ample time to reflect and answer the questions.

### 2.5. Statistical Methods

Statistical analysis was conducted using SPSS 29.0, Jamovi 2.3.18, and Office 2003 Excel. The scores of items 4, 9, 15, 17, 19, 23, and 27 (7 items) in the “Stereotypes” domain were recoded prior to statistical analysis. The recoding scheme for the Likert scale scores was as follows: 5 = 1, 4 = 2, 3 = 3, 2 = 4, 1 = 5 [2].

In the first phase, descriptive statistics of the questionnaire on the characteristics of the sample were calculated, the normality of the distributions were related to each of the items, and the total questionnaire score was verified by calculating skewness and kurtosis indices [32]. The significance of some socio-demographic variables was determined using the *t*-test.

Pearson’s correlation coefficient (r) was used to assess the degree of association between each item and the overall scale (item-to-total correlation). Acceptable values for this index (r) were considered to be greater than 0.30 [33].

Subsequently, to verify the internal consistency of the questionnaire, Cronbach’s alpha coefficient was calculated [34,35]. To evaluate the contribution of each item to the reliability of the scale, the changes in alpha values with the item deleted were determined [36].

Before proceeding with the factor analysis, tests for adequacy and sample size were conducted using the Kaiser–Meyer–Olkin (KMO) measure, accepting values > 0.60, and Bartlett’s test of sphericity, highlighting its significance [36].

Exploratory factor analysis (EFA) was conducted with two objectives as follows: instrumental reduction by eliminating unnecessary items and identification of the main factors for data reduction.

The questionnaire was analyzed with the following four sections using orthogonal varimax rotation: 1. Role and Professionalism; 2. Stereotypes; 3. Values and Advocacy; 4. Motivation and Satisfaction. Internal consistency (α), sample adequacy (KMO), and the strength of the relationship between variables (Bartlett’s test of sphericity) were calculated for all sections.

Spearman’s correlation coefficient (Rho) was used to determine the relationships between the summary variable and the research instrument.

## 3. Results

### 3.1. Demographic Characteristics of the Sample

The sample consisted of 26.4% (*n* = 149) males and 73.6% (*n* = 415) females. The age group for 34.9% (*n* = 197) was between 20 and 30 years, 20.7% (*n* = 117) were between 31 and 40 years, 22.7% (*n* = 128) were between 41 and 50 years, 14.9% (*n* = 84) were between 51 and 60 years, and 6.0% (*n* = 34) were between 61 and 70 years. Up to 51.4% (*n* = 290) had a high school diploma, 28.9% (*n* = 163) had a university degree, 11.0% (*n* = 62) had a middle school degree, 7.1% (*n* = 40) had a postgraduate degree, and 1.2% (*n* = 7) had a primary school degree. Regarding employment, 34.2% (*n* = 193) of the sample were civil servants, 18.8% (*n* = 106) were private employees, 22.7% (*n* = 128) were students, 11.0% (*n* = 62) were self-employed, 3.7% (*n* = 21) were unemployed, 4.1% (*n* = 23) were retired, and 4.1% (*n* = 23) were housewives. Approximately 46.5% (*n* = 262) have had at least one hospital admission and 71.3% (*n* = 402) have had contact with a nurse (Table 1).

Before proceeding with the calculations to determine the psychometric properties of the instrument, a *t*-test was used to calculate any statistically significant differences in the items between the citizens who have had and those who have not had at least one hospital admission. The item, “Nurses are adequately paid for the work they do”, showed a significant difference (*p* = 0.023). Both groups, although with a point < 3, expressed a mean of 2.63 ± 1.95 for the group with at least one admission versus 2.31 ± 1.293 for those who had never been hospitalized.

### 3.2. Psychometric Properties of the Instrument

The Cronbach’s alpha in the 30 items was 0.893 and varied between 0.88 and 0.89 among the factors identified in the theoretical structure of the NAQ [2]. No changes in the internal consistency reliability were found after eliminating each item one by one.

Skewness and kurtosis showed a normal distribution in the item responses in most items, and a weak tendency towards higher levels of agreement (mean score < 3.50) in the items 4, 9, 15, 17, 19 (Stereotypes domain), 18 (Role and Professionalism domain), 12, and 28 (Motivation and Satisfaction domain). With regard to these items, no statistical transformations were adopted because the skewness and kurtosis deviation were not critical and the methods used in the data analysis were not influenced by the data distribution [37].

From Table 1, it can also be observed that the item-to-total correlation index greatly exceeds 0.30 for all 30 items, indicating a high degree of correlation between each item and the overall scale (*p* ≤ 0.001). The item with the lowest value (<0.50) is item 18, “Men make good nurses,” with a correlation of 0.496.

The sample adequacy test (KMO) ranges from good (0.80–0.90) to excellent (>0.90) [38] across all items. The numbering alongside the items in Table 2 respects the original sequencing of the original instrument [2] (Table 2).

### 3.3. Exploratory Factor Analysis

The NAQ was subjected to Exploratory Factor Analysis (EFA) using the principal axis extraction method. The number of factors to be extracted was chosen based on the scree plot method [39] and the eigenvalue greater than 1.0 method [38]. In the scree plot, component 1 recorded an eigenvalue of 10.226 with a variance percentage of 34.08%, component 2 had an eigenvalue of 5.002 and a variance of 16.67%, component 3 had an eigenvalue of 1.714 and a variance of 5.71%, and component 4 had an eigenvalue of 1.219 and a variance of 4.06%.

**Figure 1:**
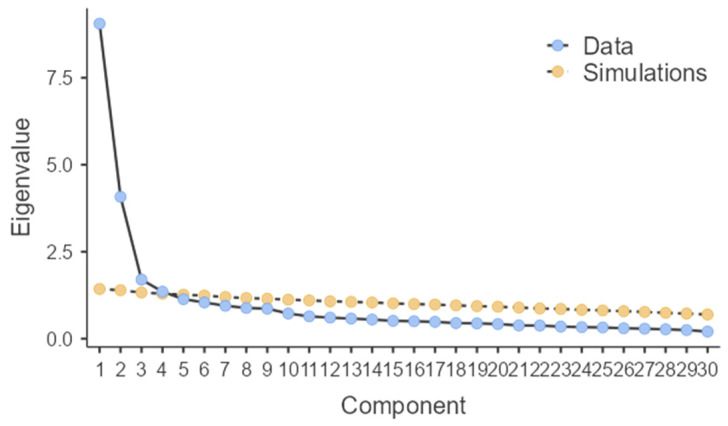
Scree plot.

Exploratory factor analysis (EFA), with varimax rotation, showed through the Scree Plot, a four-factor model showing 53.9% variance in the scale (Figure 1).

The criteria for performing the factor analysis were a verified KMO of 0.930 and the Bartlett’s sphericity test showing a *p*-value of <0.001 (chi squared = 10,786 and df = 435) [40].

There were six random eigenvalues from the parallel analysis [41,42,43] that were greater than 1.0. For the NAQ-IV, an initial four-factor solution was chosen for better grouping of items along content dimensions, as parallel analysis tends to overestimate the number of extracted factors [44].

EFA was performed using the varimax rotation method, and the correlation matrix showed correlations of more than |0.45| between most of the factors (Table 3).

### 3.4. Confirmatory Factor Analysis

Confirmatory factor analysis (CFA) seems to confirm the structure based on the data collected. The numerical sequencing of the table respects the Italian version of the tool (NAQ-IV).

The goodness-of-fit of the four-factor solution assessed in the CFA used several criteria as follows: chi squared/degrees of freedom (χ^2^/df), and root mean square error of approximation (RMSEA). The test for exact fit thus indicated an χ^2^ = 2.567 (*p* ≤ 0.001), SRMR 0.0939, RMSEA 0.0981 (IC 90% = 0.0945–0.120), TLI 0.777, and CFI 0.795.

## 4. Discussion

This study contributes to the literature by examining an Italian instrument for assessing the public image of nurses. The study revealed that participants had a positive image of nurses, as highlighted in the literature [21]. The investigated areas included the role and professionalism of nurses in socio-health contexts, stereotypes, values, and advocacy, and the motivations and satisfactions perceived by nursing professionals operating in Italy. Regarding the stereotype domain, the study showed negative correlations with the other three areas. This demonstrates the positive perception that citizens have in light of the evolving competencies of the nursing profession, which was also promoted by mass media during the pandemic period [8].

It was necessary to validate a useful tool to assess the public image of nurses in Italy to monitor how territorial policies can positively influence the attractiveness of the profession for young people [45,46], better patient satisfaction and quality of nursing care, and improved quality of work life [5,7].

The Italian version of the NAQ, named NAQ-IV, appears to be a valid instrument for investigating the perception that ordinary people have of the image of nurses. 

The psychometric characteristics of the NAQ-IV show a four-factor aggregation and good internal consistency of the items. In the past, other studies have used the NAQ by reducing the items and extrapolating factors. In 2016, Hoeve et al. [29] used the questionnaire on a population of nursing students in the Netherlands. The research aimed to evaluate the orientation and attitudes of a cohort of first-year students towards nursing at the beginning of the educational program. To this end, the authors reduced the NAQ items from 30 to 18 [47,48,49] and extrapolated two factors from the questionnaire, namely “Nursing Agency” (14 items α = 0.74) and “Advocacy and Empathy” (4 items α = 0.63). In this study, Confirmatory Factor Analysis (CFA) showed a fit with X^2^ = 3.69; CFI = 0.90; RMSEA = 0.046; α = 0.79.

From 2017 to 2023, studies were conducted in Italy using the NAQ. These studies aimed to evaluate the perception of ordinary people towards the image of nurses and nursing care. The authors focused on an overall result of the 30 items of the NAQ and on the areas named by Toth et al. in 1998. The areas indicated by Toth et al. mentioned the number of items per area in the article without clearly indicating which questions they were. Furthermore, the study did not include factor analysis calculations. For this reason, in the Italian studies, the content validity of the areas suggested by Toth et al. was supported by a panel of experts who reviewed the NAQ questions and assigned the most appropriate items for each area. No factor analysis calculations were performed, only overall internal consistency calculations and correlation coefficients between areas [21,30].

Therefore, the NAQ-IV has only one comparative study, that of Hoeve et al. [29], which, although it presented factor analysis calculations, used the NAQ with reduced items.

The NAQ-IV, although it presents 14 items in the first factor, shows no evidence of similarity with the first factor explored by Hoeve et al. [29] as their study did not report the constituent items. However, the methodology for naming the areas in the Italian version of the NAQ followed the study by Hoeve et al. [29]. A team of four researchers examined the NAQ items to verify that their formulation reflected the concept measured—the image of nurses and the attitude of the sample towards nursing. The specific meaning of each item was examined to establish face validity. The identified domains are confirmed by previous studies on the NAQ, phylogenesis, and the organizational, educational, and care models of the nursing profession. The areas “Role and Professionalism” and “Stereotypes” express the concepts highlighted by the study of Toth et al. [2] as well as Bolan and Grainger [27], while “Values and Advocacy” refers to the extent to which nurses speak and act for patients, specifically addressing unmet patient needs, making it a value for the profession to satisfy these needs [27,47,48]. Regarding nurse motivation and satisfaction, a significant intrinsic relationship between these two elements was found to improve nursing care and its related image [21].

The Cronbach’s alpha coefficient on the total NAQ-IV scale used in interviews with the general public recorded a value of 0.89, with a range from 0.88 to 0.89. These results seem consistent with previous studies where the sample consisted only of nursing students. In fact, research on Canadian students showed an alpha variation from 0.80 to 0.82 [27], while Toth et al.’s study [2] found a range from 0.75 to 0.80 [28]. Therefore, the NAQ-IV appears to be a reliable instrument for assessing the perception of the image of nurses and nursing care among the general public. Furthermore, there seems to be no indication to eliminate any item based on the loading of the EFA elements [50]. A good variability of responses was found, therefore it can be stated that the formulation of the items does not generate a preferred response towards higher or lower levels of agreement, which could confirm the assumption of variability of the Likert scale [51].

## 5. Limits

A strength of the study is the use of an online platform for the questionnaire, which allowed participants from all over the country to express their opinions. However, self-completion of a digital questionnaire did not allow us to reach citizens without smartphones or those who do not use social media. Another limitation is determined by the sample size and consequently by the sample selection method. It was not possible to randomize the participants in the study, and the sample is heterogeneous in sociodemographic characteristics, with even significant percentage differences. Regarding the instrument, further studies are recommended to evaluate its reliability, especially in the case of changes in the professional profile or the Italian socio-healthcare welfare system.

## 6. Conclusions

This study demonstrated a satisfactory psychometric property in the Italian version of the Nursing Attitudes Questionnaire. With respect to previous validation works, the NAQ-IV investigated the perception of the image of the nursing profession and nursing care on a population that was not exclusively student but heterogeneous in terms of profession, culture, and age. Specifically, the NAQ-IV structured four factors investigating the respondents’ perceived role, professionalism, stereotypes, values, advocacy, motivation and satisfaction with the nursing profession. The instrument could be useful to investigate how society perceives the role and functions of the nurse and what value they attach to the profession. In previous Italian studies, the NAQ has provided significant insights into the motivations that make the profession unattractive to young people who need to undertake education to enter the workforce [21,30,31].

The NAQ-IV could be an interesting instrument to assess the internationally perceived image and nursing care among ordinary people. An international multi-center approach could therefore be useful to confirm the theoretical framework in the structure of the NAQ-IV. For future research, longitudinal studies are recommended to monitor changes in public perception over time, particularly in response to policy changes and societal shifts. Additionally, expanding this research internationally could provide comparative insights and further validate the NAQ-IV in different cultural contexts. Practical implications include the potential use of the NAQ-IV by healthcare organizations and policymakers to assess and improve the public image of nursing, thereby enhancing the profession’s attractiveness and addressing the current nursing shortage.

In summary, the NAQ-IV is a valuable instrument for investigating the perception of the nursing profession among the general public in Italy. Its use can contribute significantly to understanding and improving the factors that influence the public image of nursing, ultimately leading to better recruitment, retention, and overall quality of nursing care. Further research and international collaboration are recommended to expand the utility and applicability of the NAQ-IV in diverse settings (“see Appendix A”).

## Figures and Tables

**Table 1:** Demographic Characteristics.

		*n* (%)
Gender		
	Males	149 (26.4)
	Females	415 (73.6)
		
Age group		
	20–30 years	197 (34.9)
	31–40 years	117 (20.7)
	41–50 years	128 (22.7)
	51–60 years	84 (14.9)
	61–70 years	34 (6.0)
	Missing	4 (0.7)
		
Occupation		
	Public employees	193 (34.2)
	Students	128 (22.7)
	Private employees	106 (18.8)
	Self-employed workers	62 (11.0)
	Retirees	23 (4.1)
	Homemakers	23 (4.1)
	Unemployed	21 (3.7)
	Missing	8 (1.4)
		
Previous hospitalizations		
	Yes	262 (46.5)
	No	87 (15.4
	Missing	215 (38.1)
	
Known nurse		
	Yes	402 (71.3)
	No	151 (26.8)
	Missing	11 (1.9)

**Table 2:** Position and shape indices, item–total correlation and Cronbach’s alpha.

NAQ—IV	M ± SD	Skewness	Kurtosis	Corrected Item to Total Correlation	Cronbach’s Alpha If Item Deleted	KMO Measure of Sampling Adequacy (MSA)
Role and Professionalism α = 0.934 (14 Items)						
5. Nurses act as resource persons for individuals with health problems	4.24 ± 0.959	−1.42	2.07	0.770 ***	0.885	0.953
7. It takes intelligence to be a nurse	4.13 ± 0.960	−1.09	1.03	0.774 ***	0.885	0.949
8. The service given by nurses is as important as that given by physicians	4.24 ± 0.970	−1.30	1.44	0.790 ***	0.884	0.948
10. Nurses integrate health teaching into their practice	4.01 ± 0.912	−0.826	0.826	0.788 ***	0.885	0.957
11. Research is vital to nursing as a profession	4.04 ± 0.984	−0.875	0.434	0.791 ***	0.884	0.944
13. Nurses are capable of independent practice	3.79 ± 1.13	−0.655	−0.265	0.694 ***	0.885	0.953
16. Nurses should have a right to strike	3.99 ± 1.02	−0.808	0.251	0.712 ***	0.886	0.967
18. Men make good nurses	3.46 ± 1.20	−0.469	−0.505	0.496 ***	0.893	0.932
20. Nursing is exciting	3.87 ± 1.01	−0.573	−0.142	0.795 ***	0.884	0.957
21. Nurses incorporate research findings into their clinical practice	3.80 ± 0.987	−0.464	−0.150	0.803 ***	0.884	0.948
22. The major goal of nursing research is to improve patient care	4.04 ± 0.995	−0.913	0.527	0.829 ***	0.884	0.946
24. Nurses value time at the bedside caring for patients	3.70 ± 1.04	−0.495	−0.076	0.725 ***	0.886	0.962
25. Nurses should have a Baccalaureate degree for entrance into practice	4.07 ± 1.02	−0.872	0.258	0.797 ***	0.884	0.946
26. Nurses with advanced degrees make important contributions to patient care	3.59 ± 1.08	−0.483	−0.136	0.621 ***	0.888	0.941
Stereotypes α = 0.865 (7 Items)						
4. Nurses should wear a white uniform in order to be identified	2.90 ± 1.28	0.136	−0.992	0.604 ***	0.898	0.874
9. Everyone would benefit if nurses spent less time in school and more time caring for patients	3.15 ± 1.33	0.009	−1.09	0.684 ***	0.898	0.868
15. Nurses are compensated sufficiently for their work by the knowledge that they are helping people	3.34 ± 1.24	−0.174	−0.879	0.747 ***	0.898	0.883
17. Nurses follow the physician’s orders without questions	3.29 ± 1.20	0.061	−0.814	0.739 ***	0.896	0.874
19. Many nurses who seek advanced degrees in nursing would really rather be physicians	3.46 ± 1.23	−0.274	−0.770	0.722 ***	0.894	0.848
23. Nurses are adequately paid for the work they do	3.63 ± 1.22	−0.403	−0.752	0.627 ***	0.894	0.876
27. One advantage to being a nurse is to marry a physician	4.09 ± 1.18	−1.02	0.054	0.770 ***	0.894	0.860
Values and Advocacy α = 0.838 (4 Items)						
1. Nurses are patient’s advocates	3.87 ± 1.05	−0.655	−0.163	0.877 ***	0.889	0.877
2. Nurses protect patients in the health care system	4.02 ± 1.00	−0.924	0.494	0.888 ***	0.887	0.883
3. Nurses participate in the development of health care policies	3.87 ± 1.05	−0.737	0.011	0.811 ***	0.889	0.940
6. Nurses in general are kind, compassionate human beings	3.58 ± 1.02	−0.531	0.103	0.707 ***	0.891	0.934
Motivation and Satisfaction α = 0.727 (5 Items)						
12. Nurses are politically active	3.29 ± 1.09	−0.302	−0.180	0.693 ***	0.892	0.921
14. Nurses speak out against inadequate working conditions	3.54 ± 1.05	−0.266	−0.262	0.677 ***	0.889	0.938
28. Nursing is a respected profession	2.90 ± 1.20	0.087	−0.732	0.660 ***	0.899	0.884
29. Nurses consistently update their practice in relation to current health trends	3.61 ± 1.04	−0.291	−0.370	0.671 ***	0.887	0.951
30. Nurses feel good about what they do	3.19 ± 1.03	−0.218	0.067	0.769 ***	0.893	0.909

*** *p* ≤ 0.001. α = 0.893, KMO = 0.930.

**Table 3:** Explorative Factor Analysis, PAF, direct varimax rotation.

	Role and Professionalism	Stereotypes	Values and Advocacy	Motivation and Satisfaction
1. Nurses act as resource persons for individuals with health problems	0.712	0.015	0.496	−0.105
2. It takes intelligence to be a nurse	0.753	−0.001	0.371	−0.088
3. The service given by nurses is as important as that given by physicians	0.754	0.101	0.397	−0.086
4. Nurses integrate health teaching into their practice	0.714	0.025	0.377	0.088
5. Research is vital to nursing as a profession	0.687	0.097	0.352	0.169
6. Nurses are capable of independent practice	0.472	0.156	0.212	0.355
7. Nurses should have a right to strike	0.673	0.135	0.176	0.110
8. Men make good nurses	0.470	−0.269	0.003	0.113
9. Nursing is exciting	0.714	0.020	0.232	0.275
10. Nurses incorporate research findings into their clinical practice	0.681	0.073	0.184	0.468
11. The major goal of nursing research is to improve patient care	0.790	0.122	0.150	0.236
12. Nurses value time at the bedside caring for patients	0.621	−0.055	0.144	0.442
13. Nurses should have a Baccalaureate degree for entrance into practice	0.840	0.109	0.023	0.130
14. Nurses with advanced degrees make important contributions to patient care	0.596	−0.155	−0.022	0.359
15. Nurses should wear a white uniform in order to be identified	−0.084	0.634	−0.087	0.309
16. Everyone would benefit if nurses spent less time in school and more time caring for patients	0.100	0.620	−0.300	0.012
17. Nurses are compensated sufficiently for their work by the knowledge that they are helping people	0.059	0.738	−0.104	−0.227
18. Nurses follow the physician’s orders without questions	0.040	0.745	−0.096	−0.015
19. Many nurses who seek advanced degrees in nursing would really rather be physicians	0.054	0.749	0.071	0.115
20. Nurses are adequately paid for the work they do	0.103	0.822	0.060	−0.104
21. One advantage to being a nurse is to marry a physician	0.261	0.756	−0.128	−0.163
22. Nurses are patient’s advocates	0.203	−0.072	0.808	0.178
23. Nurses protect patients in the health care system	0.340	−0.017	0.784	0.081
24. Nurses participate in the development of health care policies	0.270	−0.126	0.695	0.150
25. Nurses in general are kind, compassionate human beings	0.280	−0.302	0.558	0.125
26. Nurses are politically active	0.177	−0.251	0.382	0.472
27. Nurses speak out against inadequate working conditions	0.290	−0.140	0.381	0.517
28. Nursing is a respected profession	0.166	−0.243	−0.076	0.675
29. Nurses consistently update their practice in relation to current health trends	0.470	−0.012	0.223	0.533
30. Nurses feel good about what they do	0.300	−0.374	0.069	0.508
% of variance Total % of variance of the factor model	24.77	15.88	12.32	7.52 60.52

## Data Availability

Data are contained within the article.

## Appendix A

Nursing Attitudes Questionnaire—Italian Version (NAQ-IV)

Scala di valutazione:

1 = forte disaccordo; 2 = disaccordo; 3 = né accordo né disaccordo; 4 = accordo; 5 = forte accordo.

**Ruolo & Professionalità**
1. Gli infermieri rappresentano una risorsa per le persone con problemi di salute
2. Essere un infermiere/a richiede intelligenza
3. Il servizio espletato dagli infermieri è importante quanto quello fornito dai medici
4. Gli infermieri integrano gli insegnamenti sanitari nella pratica assistenziale
5. La ricerca scientifica è vitale per la professione infermieristica
6. Gli infermieri sono in grado di operare in modo autonomo
7. Gli infermieri devono avere il diritto di scioperare
8. Gli uomini sono dei bravi infermieri
9. Il lavoro di infermiere è emozionante
10. Gli infermieri integrano i risultati delle ricerche scientifiche nella pratica clinica
11. L’ obiettivo più importante della ricerca infermieristica è quello di migliorare l’ assistenza al paziente
12. Gli infermieri attribuiscono valore al tempo che trascorrono al letto dei pazienti per prendersi cura di loro
13. Gli infermieri devono avere una laurea per praticare la professione
14. Gli infermieri che hanno conseguito una laurea magistrale danno contributi importanti per la cura dei pazienti
**Stereotipi**
15. Gli infermieri devono indossare una divisa bianca per essere identificati
16. Se gli infermieri trascorressero più tempo a prendersi cura di pazienti e meno all’ università tutti ne trarrebbero beneficio
17. Gli infermieri sono ripagati sufficientemente per il loro lavoro dal sapere che stanno aiutando altre persone
18. Gli infermieri eseguono le richieste del medico senza obiezione
19. Molti infermieri che ricercano un avanzamento nella loro attività professionale, in realtà vorrebbero essere piuttosto dei medici
20. Gli infermieri sono adeguatamente pagati per il lavoro che svolgono
21. Uno dei vantaggi di essere un infermiere è quello di sposare un medico
**Valore & advocacy**
22. Gli infermieri sono coloro che sostengono i diritti dei pazienti
23. Gli infermieri tutelano i pazienti all’ interno del Sistema Sanitario
24. Gli infermieri partecipano allo sviluppo delle politiche sull’ assistenza sanitaria
25. Gli infermieri sono in genere gentili e compassionevoli
**Motivazione & Soddisfazione**
26. Gli infermieri sono politicamente attivi
27. Gli infermieri pronunciano nettamente contro condizioni inadeguate di lavoro
28. Il lavoro di infermiere è una professione rispettata
29. Gli infermieri aggiornano costantemente la loro pratica in relazione alle ultime scoperte sull’ assistenza sanitaria
30. Gli infermieri sono soddisfatti del lavoro che svolgono I punteggi degli item 4, 9, 15, 17, 19, 23, 27 (7 item) dell’area “Stereotypes” (tabella 1) devono essere ricodificati prima prima dell’analisi statistica. La modalità di ricodifica sui punteggi likert deve essere: 5 = 1; 4 = 2; 3 = 3; 2 = 4; 1 = 5.
